# Central retinal artery occlusion – detection score

**DOI:** 10.3389/fmed.2023.1129002

**Published:** 2023-03-01

**Authors:** Maria Casagrande, Nils Alexander Steinhorst, Susanne Kathrin Dippel, Fabian Kück, Carsten Grohmann, Martin S. Spitzer, Sven Poli, Nicolas Feltgen, Maximilian Schultheiss

**Affiliations:** ^1^Department of Ophthalmology, University Medical Center Hamburg-Eppendorf (UKE), Hamburg, Germany; ^2^Department of Ophthalmology, University Medical Center Göttingen, Göttingen, Germany; ^3^Department of Medical Statistics, University Medical Center Göttingen, Göttingen, Germany; ^4^Department of Neurology and Stroke, University of Tübingen, Tübingen, Germany; ^5^Hertie Institute for Clinical Brain Research, University of Tübingen, Tübingen, Germany

**Keywords:** central retinal artery occlusion, CRAO, stroke, questionnaire, triage

## Abstract

**Purpose:**

To investigate the sensitivity and specificity of central retinal artery occlusion (CRAO)-Detection Score in diagnosing CRAO *via* questionnaire and without fundoscopy.

**Methods:**

This prospective study enrolled 176 emergency patients suffering from acute visual loss, of whom 38 were suffering from CRAO. Before conducting any examination, we administered our questionnaire containing six questions, followed by a thorough ophthalmologic examination to make the diagnosis. Statistical analysis involved a LASSO penalised multivariate logistic regression model.

**Results:**

Our receiver operating characteristic (ROC) analysis based on a LASSO penalised multivariate logistic regression model showed an area under the curve (AUC) of 0.9 – three out of six questions were selected by LASSO. Interestingly, the unweighted ROC analysis of only two questions (Short CRAO-Detection Score) yielded similar results with an AUC of 0.88. The short CRAO-Detection Score of 2 yielded 14% (4/28) false positive patients.

**Conclusion:**

This prospective study demonstrates that a high percentage of CRAO patients are detectable with a questionnaire. The CRAO-Detection Score might be used to triage patients suffering acute visual loss, which is important as intravenous fibrinolysis seem to be time-dependent to be effective.

## 1. Introduction

Central retinal artery occlusion (CRAO) is an ophthalmological emergency causing severe, permanent vision loss in 95% of patients (1). Cerebral ischemia coincides with the CRAO in approximately 30% of patients, ranging from 19.5 to 37% (2–5).

There is currently no evidence-based therapy for CRAO, but intravenous thrombolysis (IVT) within 4.5 h after symptom onset has been investigated in prospective randomized controlled trials (6, 7). Trials and metanalyses have demonstrated promising results with a visual recovery in 37.3–43.8% of cases (7–10).

A major problem though is that patients suffering from CRAO often present too late for IVT (11, 12). This may be caused by insufficient awareness among the general public and health care services and the need for an ophthalmologist for diagnosis.

CRAO is usually a clinical diagnosis, but not every stroke unit offers fundoscopy. Over half of the stroke units in Germany (51.9%) reported a lack of ophthalmologic expertise (13). Furthermore, fundoscopic anomalies, such as a cherry red spot or visible retinal pallor, may be lacking within the first hours after symptom onset (9), which is why alternative diagnosing tools and biomarkers for retinal ischemia are being investigated (14–19). All diagnosing tools not involving fundoscopy may enhance the chances of CRAO patients being treated within 4.5 h after symptom onset. Patients suspected of having CRAO may be identified and then given higher priority.

To triage patients with acute visual loss and to identify CRAO patients, we developed a CRAO-Detection Score consisting of six questions (20). The purpose of this prospective study was to determine this CRAO-Detection Score’s sensitivity and specificity in two tertiary care facilities on ophthalmologic emergency patients. To the best of our knowledge, ours is the first such questionnaire to identify CRAO patients.

## 2. Materials and methods

### 2.1. Study design

A two-center, prospective clinical study with blinded investigators was conducted. Institutional Review Board approvals were obtained from the independent ethics committees (Federal Republic of Germany: States of Hamburg: PV5867; Lower-Saxony: 14/4/19Ü). All investigations were conducted according to the principles of the Declaration of Helsinki. Written informed consent was obtained from every patient prior to this survey.

### 2.2. Questionnaire

We derived a questionnaire by relying on the cardinal CRAO symptoms and potential differential diagnosis (Table 1) (20). The questionnaire consisted of six questions, which were especially important for CRAO patients undergoing IVT. The answers indicating suspicious for non-ischemic CRAO are marked in gray.

**Table 1:** Six questions derived from the characteristic symptoms of CRAO and its differential diagnoses.

1. Did your severe loss of visual acuity occur within seconds?	YES	NO
2. Was your visual acuity in the affected eye normal prior to the incident?	YES	NO
3. Did you notice the visual loss noticed with both eyes open?	YES	NO
4. Is your entire visual field consistently dark-shadowed?	YES	NO
5. Do you feel any pain in or around the affected eye?	YES	NO
6. Did you suffer headaches or pain while chewing and combing during the last few days?	YES	NO

“Gray” fields show a pattern, that might indicate a possible CRAO.

### 2.3. Setting

This study was conducted at the Eye Hospital of two tertiary care facilities (Universitätsklinikum Eppendorf/Hamburg; Universitätsmedizin Göttingen/Lower-Saxony). Over the course of 17 months, 176 emergency patients were enrolled in the study.

The inclusion criteria were adult patients above 18 years old presenting with subjective acute visual loss in one eye. Excluded were patients with a positive SARS-CoV-2 test, without sufficient language skills, dementia, or other cognitive impairment, and patients under 18 years of age.

This questionnaire was administered to the patients prior to any examination. After answering the questionnaire, every patient underwent a thorough ophthalmologic examination. The ophthalmologic diagnosis was written on the anonymised questionnaire and collected for statistical analysis.

### 2.4. Statistical analysis

Each answer was coded by “1” (corresponding to gray fields in Table 1) or “0.”

Fisher’s-Exact-Test and the Mann–Whitney-U-Test were used for group comparisons.

A multivariate logistic regression model was fitted to determine an optimal risk score using the least absolute shrinkage and selection operator (LASSO) method (21) for automated variable selection and regularization. We included the center in the model to control for any center differences. The optimal regularization parameter λ was determined *via* five-fold cross-validation using the “one-standard-error” rule (22). To obtain a simpler score we also raised the value of the regularization parameter λ so that exactly two variables (questions) were selected, and we considered the sum of the unweighted answers to these two questions as an alternative score (Short CRAO Detection Score). Receiver operating characteristic (ROC) analyzes were performed for both scores. The model quality was assessed by computing the associated area under the curve (AUC) and corresponding 95% DeLong confidence intervals. Identification of a possible overfit to the dataset of the penalized logistic regression model was accomplished *via* five-way stratified cross-validation.

For statistical testing, the level of significance was set to 5%.

The analysis was carried out using the statistical programming environment R/Gnu S (version 3.6.2; R Core Team 2019) using the R packages glmnet (version 4.1.2), ROCR (version 1.0.7), pROC (version 1.18.0), and cvAUC (version 1.1.0).

## 3. Results

### 3.1. Patient population

Between April 2020 and August 2021, 176 patients with acute visual loss were enrolled into this prospective study (Göttingen: 112, Hamburg 64). Of these patients, *n* = 38 were suffering from CRAO and 138 from 13 other pathologies: anterior ischemic optic neuropathy *n* = 13; retinal detachment *n* = 20; branch retinal artery occlusion *n* = 19; posterior vitreous detachment *n* = 7; vitreous hemorrhage = 12; glaucoma attack *n* = 5; corneal affection *n* = 8; cataract *n* = 7; intraocular lens luxation *n* = 5; macula hemorrhage *n* = 17; migraine ophthalmic *n* = 2; branch retinal vein occlusion *n* = 11; and central retinal vein occlusion *n* = 12.

### 3.2. Central retinal artery occlusion-detection score

Concerning each question, only the questions 1 [Did your severe loss of visual acuity occur within seconds? (*p* < 0.001)], 2 [Was your visual acuity in the affected eye normal prior to the incident? (*p* = 0.001)], and 4 [Is your entire visual field consistently dark-shadowed? (*p* < 0.001)] yielded significantly higher mean scores from CRAO-cohort than the differential diagnoses (Table 2).

**Table 2:** Absolute and relative frequencies of positive answers for CRAO patients and patients with other diagnoses.

Question	CRAO	Other diagnoses	*p* value
Question one	32 (84.2%)	42 (30.4%)	<0.001
Question two	38 (100.0%)	110 (79.7%)	0.001
Question three	34 (89.5%)	105 (76.1%)	0.113
Question four	27 (71.1%)	9 (6.5%)	<0.001
Question five	37 (97.4%)	122 (88.4%)	0.126
Question six	34 (89.5%)	129 (93.5%)	0.482

**Figure 1:**
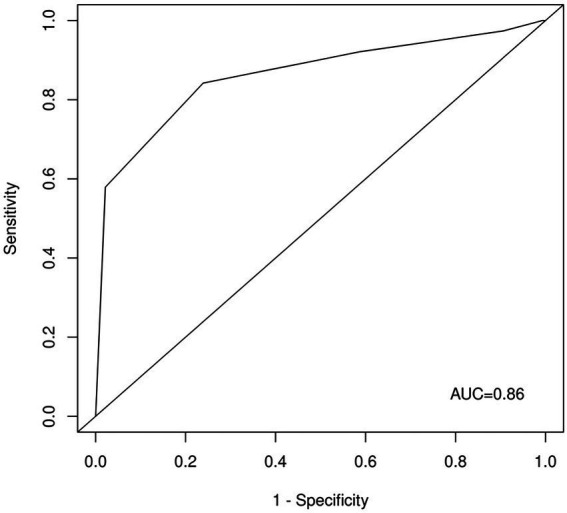
ROC curve for questions one, two, three, four, five, and six. AUC-area under the curve.

The ROC analysis of the unweighted sum of answers to questions 1, 2, 3, 4, 5, and 6 (Figure 1) showed an AUC of 0.86. A cutoff of 4 showed 0.92 sensitivity, 0.41 specificity, a positive predictive value of 0.3, and a negative predictive value of 0.95. A cutoff of 6 showed 0.58 sensitivity, 0.98 specificity, a positive predictive value of 0.88, and a negative predictive value of 0.89.

**Figure 2:**
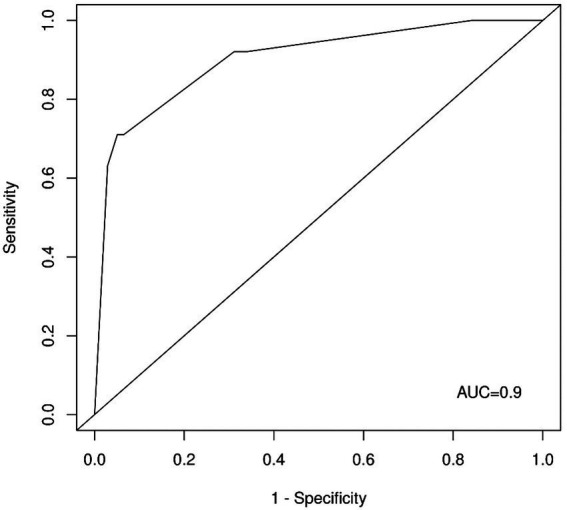
ROC curve for the LASSO regression score. AUC–area under the curve.

In a penalized multivariate logistic regression model with variables automatically selected by LASSO with the optimal regularization parameter (*λ* = 0.039) as a variable selection and weighting method, question four (*β* = 2.46) revealed the strongest positive predictive potential to detect CRAO patients, followed by question one (*β* = 1.08), and question two (β = 0.09). The corresponding receiver operating characteristic (ROC) analysis (Figure 2) showed an area under the curve (AUC) of 0.90 (95% CI: 0.85–0.96). The AUC after cross-validation was 0.89 (95% CI: 0.80–0.97).

### 3.3. Short CRAO-detection score

**Figure 3:**
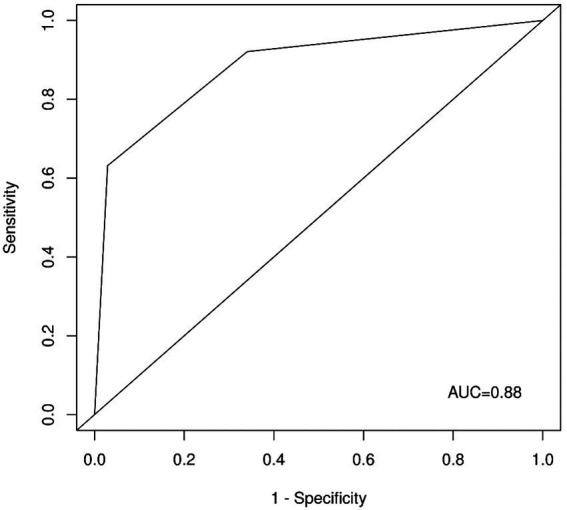
ROC curve for questions one and four. AUC-area under the curve.

Interestingly, the ROC analysis of the unweighted sum of the answers to questions one and four (Figure 3) delivered comparable results with an AUC of 0.88 (95% CI: 0.83–0.95). Note that these two questions were selected by LASSO if we chose a larger regularization parameter, meaning that only two variables were selected (e.g., *λ* = 0.061). As a triage tool, the CRAO-Detection Score should be easy to apply and should be applied as quickly as possible. Therefore, in the subsequent analyzes, we focused on the Short CRAO-Detection Score consisting of unweighted questions one and four.

A cutoff of two in the Short CRAO-Detection Score showed 0.63 sensitivity, 0.97 specificity, a positive predictive value of 0.86, and a negative predictive value of 0.91. A cutoff of 1 in the Short CRAO-Detection Score showed 0.92 sensitivity, 0.66 specificity, a positive predictive value of 0.43, and a negative predictive value of 0.97.

We noted a score of 2 existed in 63,2% (24/38) of CRAO patients and in 2.9% (4/138) of patients with differential diagnoses. We observed false positive patients with a score of 2 occurred in patients suffering from retinal detachment, branch retinal artery occlusion, vitreous hemorrhage, and glaucoma attack.

We noted a score of ≥ 1 existed in 92% (35/38) of CRAO patients and in 34.1% (47/138) of those with differential diagnoses.

The ROC analysis revealed only minor differences between the two tertiary care facilities. The AUC calculated for each center was 0.86 for Göttingen and 0.94 for Hamburg.

## 4. Discussion

This prospective study demonstrated that a high percentage of CRAO patients can be detected by asking only two specific questions. If only one positive answer in the Short CRAO-Detection Score is taken as the cutoff, the sensitivity is very high at 0.92 and the specificity still reveals a respectable 0.66. Importantly, with 2 as your cutoff, the specificity and the positive predictive value rises to 0.97 and 0.86, respectively. Therefore, the short CRAO-Detection Score seems to be well suited for prehospital screening and to potentially accelerate the prehospital procedures.

Only 2.9% in our differential-diagnosis group were false positive patients with a score of 2 in the Short CRAO-Detection Score. As the positive predictive value was 0.86 (24/28), it is much more likely that patients with a score of 2 are suffering from a CRAO. Note that 23% of CRAO patients had a score of 1. Therefore, patients scoring 2 should be immediately transferred to a tertiary care facility with a stroke unit. Patients with a score of 1 should undergo an ophthalmologic examination as soon as possible.

Since achieving these very promising results, we will employ this questionnaire in the REVISION-Trial [Early REperfusion Therapy with Intravenous Alteplase for Recovery of VISION in Acute Central Retinal Artery Occlusion (REVISION), a double-blind randomized placebo-controlled phase II proof-of-concept trial; ClinicalTrials.gov Identifier: NCT04965038]. After the REVISION-Trial, data on the robustness of the questionnaire will be available. Furthermore, as the REVISION-Trial will be investigating the biomarkers observed in ocular coherence tomography (14, 17), it should be possible to diagnose CRAO without fundoscopy in the future. Diagnosing CRAO without fundoscopy would be especially important in stroke units, which frequently lack the ophthalmologist expertise to confirm CRAO, keeping in mind that the effectivity of IVT seems to be extremely time-dependent (9, 10). Therefore, such an alternative workflow (questionnaire plus ocular coherence tomography) would speed up the processing of CRAO patients and the IVT availability, as more centers could perform IVT. Nevertheless, from our point of view, based on the data available so far, an ophthalmological examination is mandatory before performing any IVT.

The Short CRAO-Detection Score demonstrated great specificity as a tool to triage patients suffering from acute visual loss. However, before applying IVT, we wish to emphasize the importance of administering the whole questionnaire, because relying on only the Short CRAO-Detection Score might result in the loss of important information. For example, genuine visual recovery must be possible to justify what is a potentially life-threatening therapy (question two). To define the precise time of symptom onset, question three is important, because if patients are only asked “when did you notice the visual loss?,” they might state the time they noticed (i.e., with one eye closed) and not the time of CRAO-onset. This is especially important in cases of CRAO when the patient has just woken up or when the visual loss is only noticed once they have covered the unaffected eye. Questions five and six exclude important differential diagnoses (e.g., giant cell arteritis). Nevertheless, the LASSO regression identified questions four (“Is your entire visual field consistently dark-shadowed?”) and one (“Did the severe loss of visual acuity occur within seconds?”) as the most important questions to identify CRAO patients. The setting where the patient with visual loss goes for medical care, such as a private ophthalmological practice or a stroke unit, might influence which questions are posed.

Among the limitations of this study was a possible selection bias as only emergency patients suffering from acute visual loss were included in our analysis. Due to the relatively low sample size, separate development and validation cohorts were not implemented. In future studies, this should be implemented to further support the data. Although the relatively low number of patients limits the validity of our (Short) CRAO-Detection Score, statistical analysis nonetheless yielded promising results in our cohort, and we observed only minor differences between the two centers through the ROC analysis. Therefore, our data can be considered robust.

In conclusion, the (Short) CRAO-Detection Score is a potentially very helpful tool to triage patients suffering from acute visual loss. Its use might lead to faster detection of CRAO patients, thus enhancing the proportion of patients presenting within the 4.5 time window to perform IVT in acute CRAO patients. Furthermore, the CRAO Detection Score covers all important questions that are essential to decide on IVT, and it therefore could be applied as part of a structured anamnesis in patients suffering from acute CRAO.

## Data availability statement

The raw data supporting the conclusions of this article will be made available by the authors, without undue reservation.

## Ethics statement

The studies involving human participants were reviewed and approved by Ethics committee Hamburg: Weidestraße 122b, 22083 Hamburg, Ethics committee Lower-Saxony: Karl-Wiechert-Allee 18–22, 30625 Hannover. The patients/participants provided their written informed consent to participate in this study.

## Author contributions

MSc, NF, SP, and MSp contributed to the conception and design of the study. MC and MSc wrote the first draft of the manuscript. FK performed the statistical analysis. NS, CG, and SD conducted the data collection. All authors contributed to the manuscript revision, read, and approved the submitted version.

## Conflict of interest

The authors declare that the research was conducted in the absence of any commercial or financial relationships that could be construed as a potential conflict of interest.

## Publisher’s note

All claims expressed in this article are solely those of the authors and do not necessarily represent those of their affiliated organizations, or those of the publisher, the editors and the reviewers. Any product that may be evaluated in this article, or claim that may be made by its manufacturer, is not guaranteed or endorsed by the publisher.
